# Pyrethroid Ingestion With Brain and Heart Involvement: A Case Report

**DOI:** 10.7759/cureus.82748

**Published:** 2025-04-21

**Authors:** Mohammed Abdurabu, Waleed Salem

**Affiliations:** 1 Emergency Medicine, Hamad Medical Corporation, Doha, QAT; 2 Emergency Medicine, Hamad General Hospital, Doha, QAT

**Keywords:** brain, heart, pesticides, pyrethroid ingestion, toxicology

## Abstract

Insecticides have great efficacy and are used in homes and agricultural fields across the globe, including pyrethrins and their synthetic equivalents, or pyrethroids. Among their compounds are deltamethrin and imiprothrin, which are present in many insecticide sprays. Humans can become toxically exposed to them through ingestion, skin contact, or inadvertent inhalation. In humans, gastrointestinal and neurotoxic side effects are frequently observed in cases of acute oral intoxication. Although they have not been well documented up to this point, pyrethroids have occasionally caused cardiotoxic consequences after consumption. This case describes a deliberate instance of pyrethroid ingestion that resulted in acute symptoms such as persistent tachycardia and a reduction in the level of consciousness. The patient underwent intensive care follow-up with a favorable outcome. Comparing our case with the available literature highlighted how rare cardiac involvement is in pyrethroid toxicity and emphasized the need for further research to verify the exact mechanism of its effects.

## Introduction

Pyrethroids are one of the most common insecticides used to control the pest population, especially in the agricultural sector. They have gained popularity due to their good efficacy and lower toxicity compared to organophosphorus compounds [1]. Over the years, pyrethroids have been developed, and many synthetic derivatives have been produced, including cypermethrin and deltamethrin. This insecticide targets the voltage-dependent sodium channels of insects, leading to excitatory paralysis of the cells and eventually resulting in death within seconds to minutes [2]. In this case report, we present a case of a young gentleman who had intentional ingestion of pyrethroid derivatives and exhibited an atypical presentation.

## Case presentation

A 24-year-old Indian gentleman with no past medical history was brought in by emergency medical services (EMS) with a suspected suicide attempt.

Upon arrival at the scene, the ambulance crew assessed the patient and noted vital signs indicative of tachycardia, while both blood pressure and body temperature remained within normal limits. The patient demonstrated a Glasgow Coma Scale (GCS) score of 14 out of 15, exhibited spontaneous movement of all four limbs, and was dry to the touch, with no apparent signs of external trauma.

History reported by a bystander indicated that the patient had ingested 400 mL of insect killer, which contained deltamethrin and cypermethrin, just a short while before the crew's arrival.

The patient was shifted to the ambulance, and en route, his GCS dropped to 6. It was decided to intubate him prior to arrival at the emergency department (ED).

**Figure 1:**
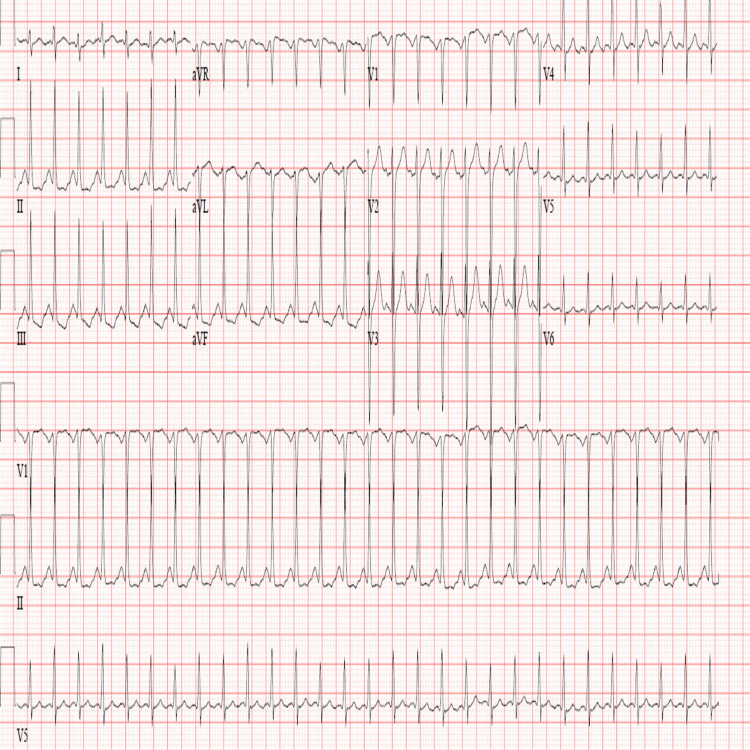
The initial ECG, which shows sinus tachycardia with non-specific ST-T wave changes

In the ED, full personal protective equipment (PPE) was worn, and skin and eye decontamination was performed. The patient was intubated and sedated, with a heart rate of 180, and the rest of the examination was unremarkable. Toxicology was consulted, and they advised supportive management, including rehydration, airway, breathing and circulation support, and neurological vital signs observation. The initial electrocardiogram (ECG) (Figure 1) showed sinus tachycardia.

**Figure 2:**
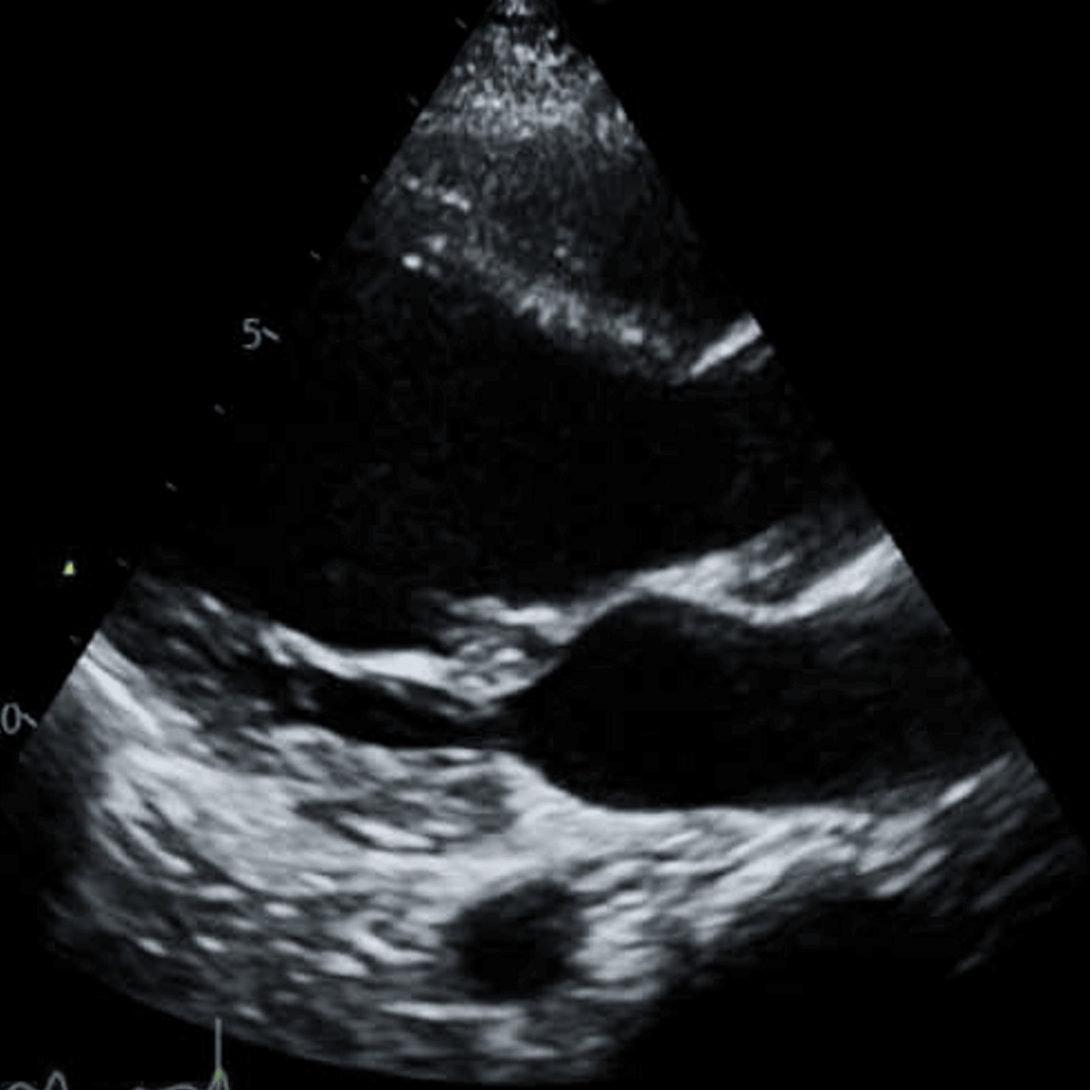
The bedside echo in its parasternal long axis view, which shows normal sized heart with fair contractility

Bedside initial echocardiogram (echo) (Figure 2) showed a normal-appearing heart with a collapsible inferior vena cava (IVC) at that time, and a 500 mg bolus of normal saline was given.

**Figure 3:**
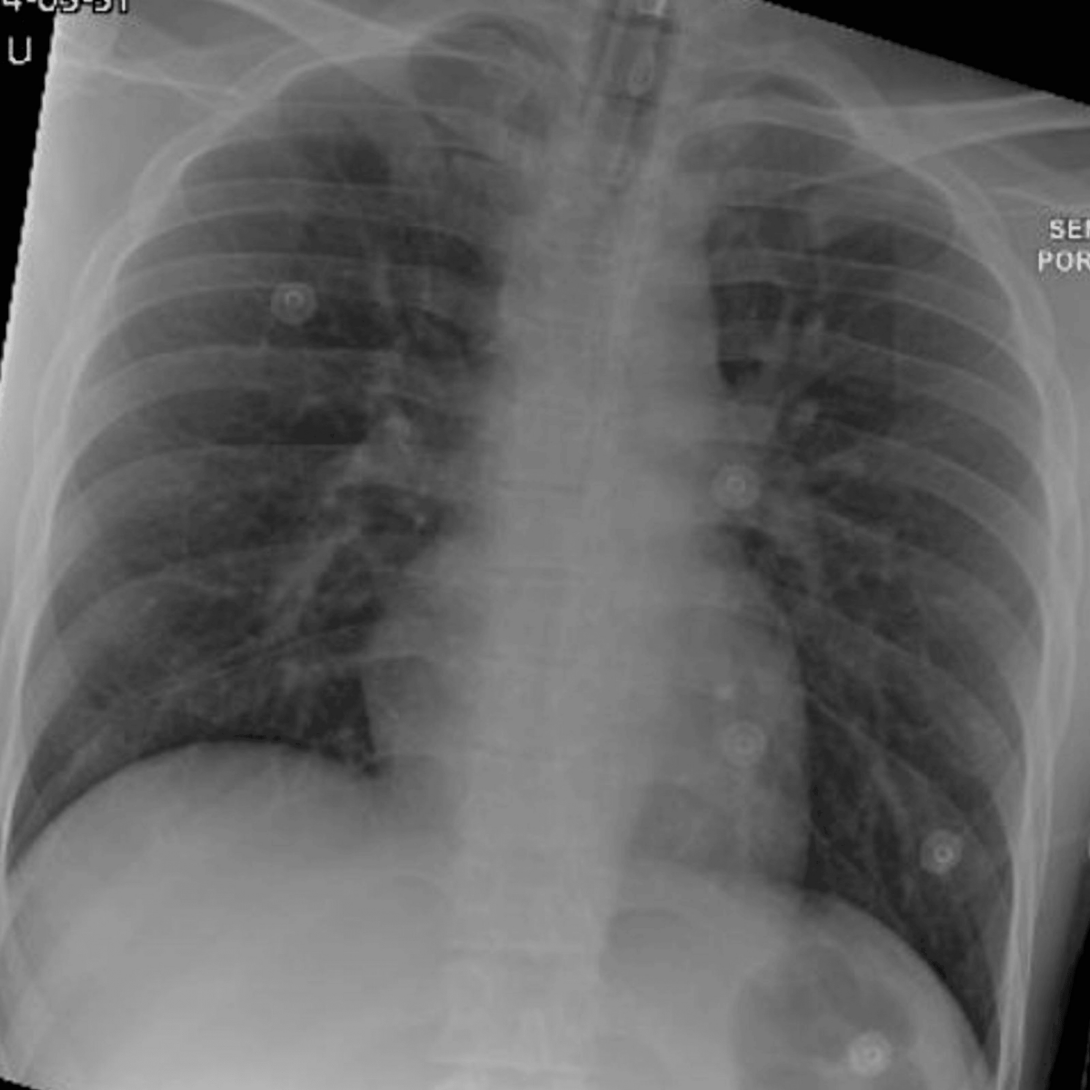
Chest X-ray, which shows an increase in the broncho vascular markings

Initial chest X-ray (Figure 3) showed increased bronchovascular markings, which were likely due to pulmonary edema, and initial CT of the head was unremarkable.

**Figure 4:**
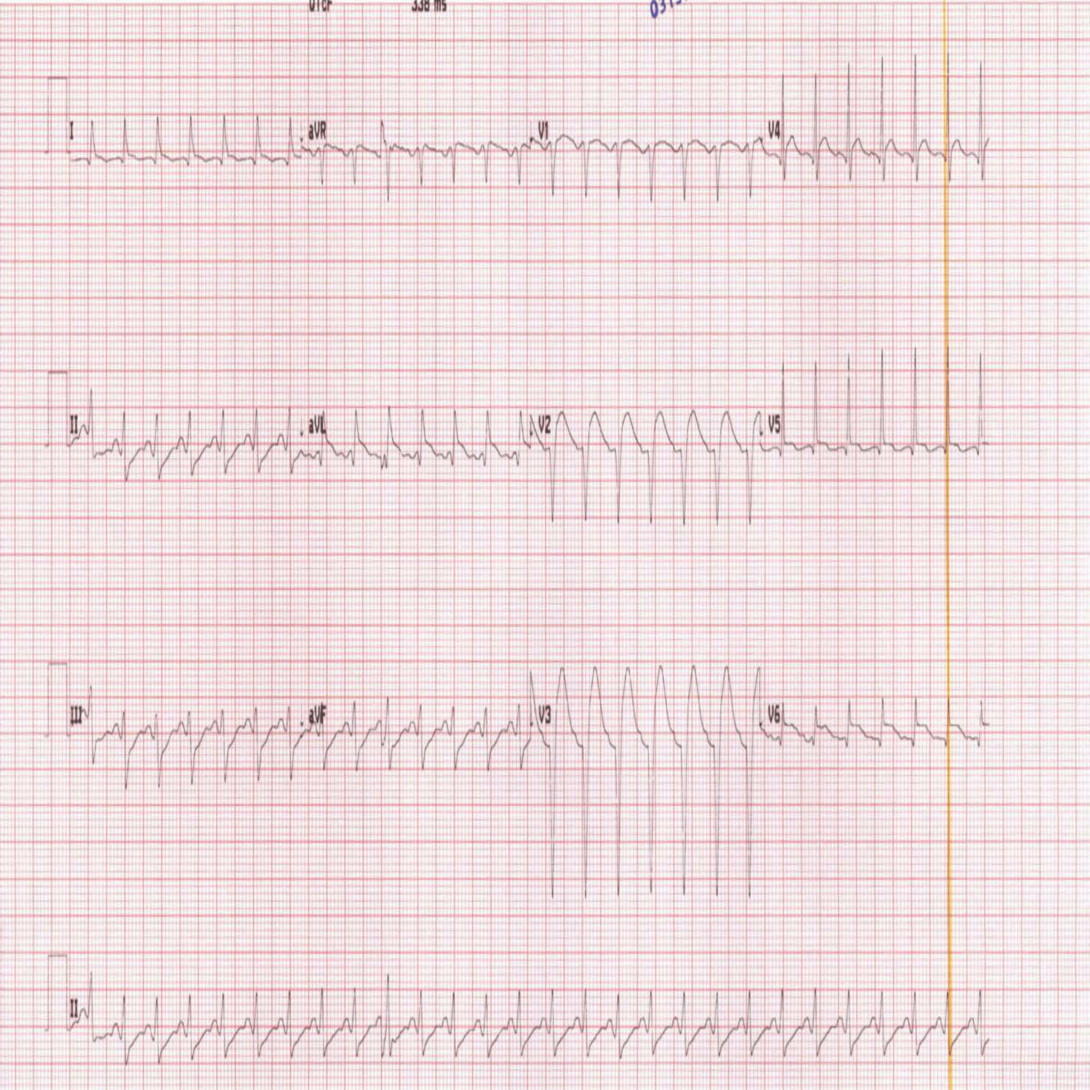
The second ECG, which shows persistent sinus tachycardia with ST elevation in the anterolateral leads

Initial blood investigations revealed elevated levels of creatine kinase, myoglobin, and troponin, while the remaining laboratory parameters were within normal limits. The medical intensive care unit (MICU) was consulted for admission. When the patient was seen by the MICU team, he developed worsening tachycardia (heart rate in the 170s) and hypertension. The ECG (Figure 4) monitor showed ST elevation in the anterolateral leads, with an increase in Troponin T from 12 to 800.

**Figure 5:**
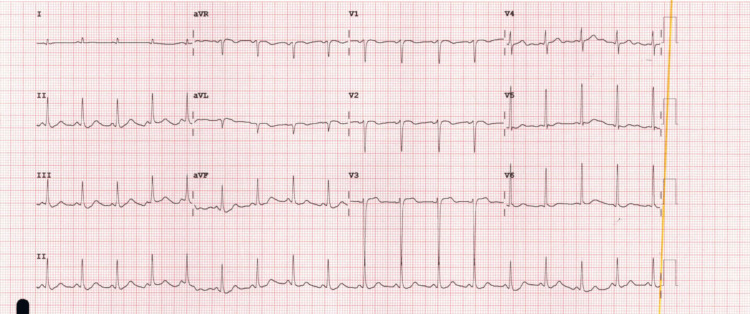
The third ECG recorded a few hours after the second ECG, which shows regression of the ST elevation and settling of the heart rate

The cardiology team was consulted, and they stated that this event was most likely caused by a spasm secondary to a metabolic cause, not occlusive coronary artery disease. They advised continuing medical treatment with aspirin and clopidogrel, with no immediate intervention. Repeat ECG (Figure 5) showed improved ST elevation after medical management.

**Figure 6:**
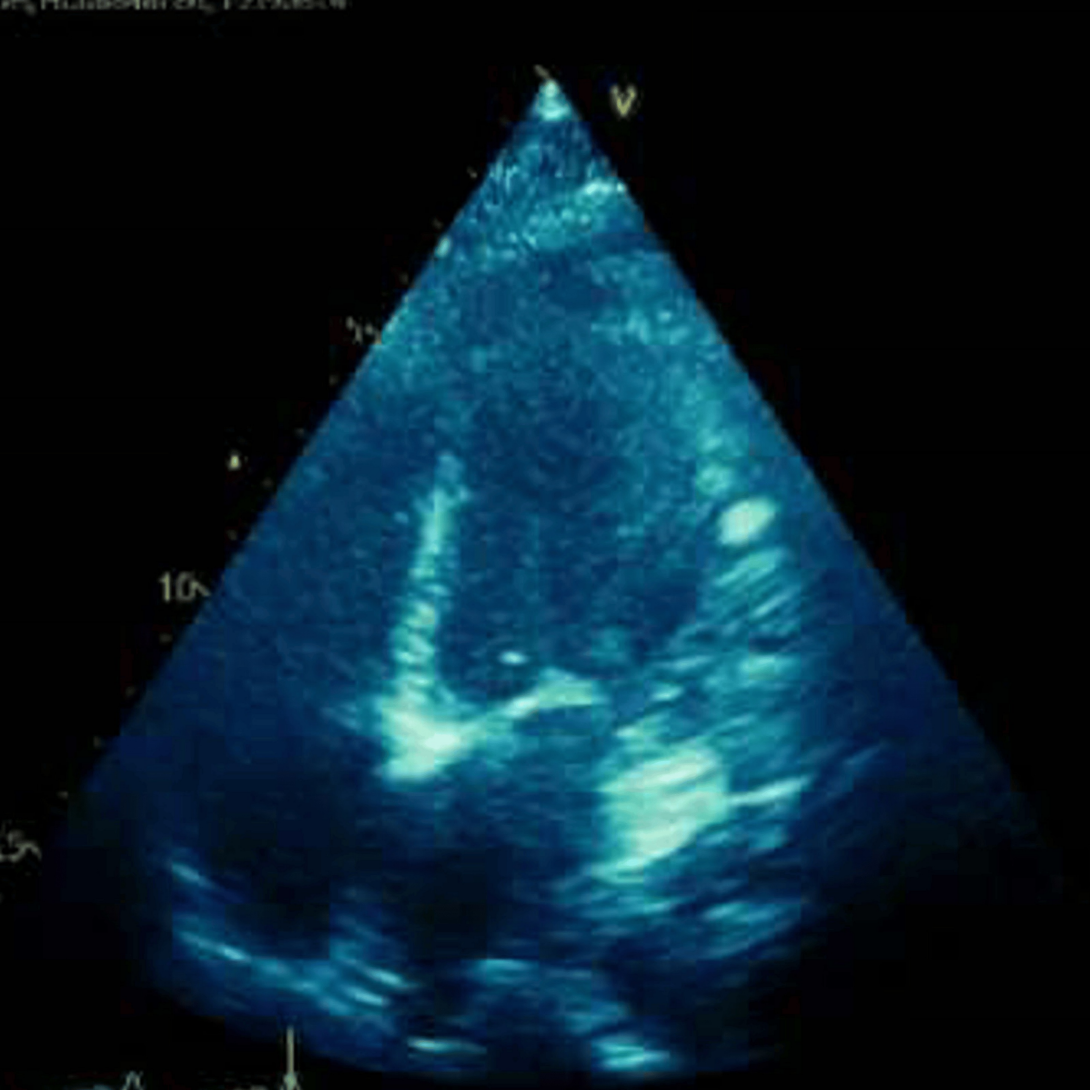
The second echo in its four-chamber view shows mild enlargement of the left ventricle with reduced ejection fraction

On the second day of admission, an official echocardiogram (Figure 6) was conducted, which showed an ejection fraction (EF) of 31%, with global basal hypokinesia and normal apical contractility, suggestive of reverse Takotsubo cardiomyopathy.

On the third day of admission, the patient was more awake and had decreasing mechanical ventilation (MV) settings along with reduced vasopressor requirements. The patient was subsequently extubated.

In the following days, psychiatry was consulted, and they recommended admission once medically stable for a full assessment with suicidal precautions.

On the seventh day of admission, the patient had returned to his baseline and was discharged with regular follow-up planned with cardiology and psychiatry.

## Discussion

Pyrethroids are classified into two types according to their chemical structure and toxidromes. Type I, such as permethrin, causes T syndrome, which is characterized by reflex hyperexcitability, paraesthesia, and fine tremors. Type II pyrethroids, such as imiprothrin, deltamethrin, and cypermethrin, are associated with the development of cholinergic syndrome, which may present with clinical features including excessive salivation, coarse tremors, seizures, and involvement of both skeletal and cardiac muscle function. A case was reported involving cypermethrin ingestion that led to severe bradycardia, which was treated with atropine [3]. When we compared our case with this report, we found contradicting findings in which our patient had persistent tachycardia despite withholding atropine and was later found to have Takotsubo cardiomyopathy. This highlights the varied and atypical cardiac effects of synthetic pyrethroids.

Ramchandra et al. conducted a comprehensive review on pyrethroid poisoning and stated that acute toxicity is generally non-life-threatening unless large doses are ingested, and that symptoms of cholinergic drive are usually predominant, attributed to the organophosphorus component of the pyrethroid molecule [4]. A retrospective study conducted in Korea involving 59 patients treated for suspected pyrethroid poisoning [5] observed atypical clinical presentations; notably, none of the patients exhibited cardiac involvement, an observation that contrasts with the presentation seen in our patient.

Bala et al. reported another case of cardiac toxicity in a 22-year-old female who intentionally ingested pyrethroids. She developed supraventricular tachycardia that was resistant to medications, with no specific cause found for the arrhythmia [6]. This case bears some resemblance to ours in terms of the presence of tachycardia; however, the underlying etiology differs. In our case, cardiac involvement was attributed to Takotsubo cardiomyopathy.

Bong et al. were involved in the management of an elderly gentleman who presented with accidental pyrethroid ingestion. Initial ECG showed atrial fibrillation, followed by the development of neurological symptoms. Subsequent imaging confirmed cerebral infarction, most likely from a cardiac-origin embolus [7]. Yet another case with cardiac arrhythmia suggests a possible link between pyrethroids and cardiac toxicity that is rarely reported.

Akelma et al. reported a case of a 25-year-old who had ingested cypermethrin and presented early with low GCS. She was admitted to the ICU for supportive management. Despite all efforts, her GCS continued to decline, which led to her being intubated for almost three days with continuous monitoring before eventual extubation [8]. Compared with our case, it is evident that central nervous system (CNS) effects are almost always predominant, with visible variation in the timing of deterioration and recovery.

## Conclusions

Pyrethroid poisoning is a rising toxicity that is rarely reported and studied. Our case presented with unique symptoms of Takotsubo cardiomyopathy when compared to the available literature. This paper discusses the rarity of some of the symptoms of pyrethroid toxicity and raises awareness about the adverse and atypical presentations of pyrethroid poisoning. We recommend that high-evidence studies, such as systematic reviews, be conducted to find out more about pyrethroid toxicity.
